# Dietary antioxidant status indices may not interact with CETP Taq1B polymorphism on lipid profile and severity of coronary artery stenosis in patients under coronary angiography

**DOI:** 10.1002/fsn3.3815

**Published:** 2023-11-28

**Authors:** Azam Ahmadi Vasmehjani, Seyed Mostafa Seyed Hosseini, Sayyed Saeid Khayyatzadeh, Farzan Madadizadeh, Mahta Mazaheri‐Naeini, Mahdie Yavari, Zahra Darabi, Sara Beigrezaei, Marzieh Taftian, Vahid Arabi, Maryam Motallaei, Amin Salehi‐Abargouei, Azadeh Nadjarzadeh

**Affiliations:** ^1^ Research Center for Food Hygiene and Safety, School of Public Health Shahid Sadoughi University of Medical Sciences Yazd Iran; ^2^ Department of Nutrition, School of Public Health Shahid Sadoughi University of Medical Sciences Yazd Iran; ^3^ Afshar Hospital Yazd Cardiovascular Research Center, Non‐Communicable Diseases Research Institute Shahid Sadoughi University of Medical Sciences Yazd Iran; ^4^ Center for healthcare Data modeling, Departments of biostatistics and Epidemiology Shahid Sadoughi University of Medical Sciences Yazd Iran; ^5^ Department of Medical Genetics, Faculty of Medicine Shahid Sadoughi University of Medical Sciences Yazd Iran; ^6^ Division of Genetics, Department of Cell and Molecular Biology and Microbiology, Faculty of Science and Biotechnology University of Isfahan Isfahan Iran

**Keywords:** antioxidants, CETP, coronary angiography, lipid profile, polymorphism, single nucleotide

## Abstract

The association of CETP Taq1B polymorphism with some metabolic traits is still controversial. The interaction of adherence to dietary indices with this polymorphism on the severity of coronary artery stenosis and serum lipid parameters needs to be investigated. This study aimed to test this hypothesis. This cross‐sectional study included 453 patients who were referred from Afshar Hospital of Yazd and undergoing coronary angiography from 2020 to 2021. Dietary intake was evaluated by a 178‐item validated and reliable dietary questionnaire. Dietary indices such as dietary antioxidant index (DAI), dietary antioxidant quality score (DAQS), and dietary phytochemical index (DPI) are determined according to dietary guidelines. The Taq1B variant was genotyped by the polymerase chain reaction‐restriction fragment length polymorphism method (PCR‐RFLP). Two‐way ANOVA was used to test the interaction between Taq1B polymorphism and dietary indices. The results of the frequency analysis of Taq1B genotypes showed that 10.4% were B1B1, 72.4% B1B2, and 17.2% B2B2. No significant interaction was found between the Taq1B variant with high adherence to DAQS, DAI, and DPI on total cholesterol (TC), high‐density lipoprotein cholesterol (HDL), low‐density lipoprotein cholesterol (LDL), triglyceride (TG) levels, and Gensini score (GS) and Syntax score (SS). In high‐adherence dietary indices, lipid profile and coronary artery stenosis scores did not differ significantly in Taq1B genotypes. Due to the insignificant results in this research, further studies are needed to investigate the role of Taq1B SNP in modulating dyslipidemia and the severity of the CAD in interaction with dietary indices.

## Graphical abstract

Our study aimed to test this hypothesis of whether adherence to dietary indices interacted with Taq1B polymorphism on the severity of coronary artery stenosis and serum lipid parameters.

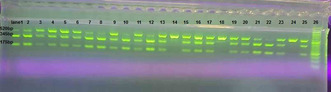

## 1 INTRODUCTION

Coronary artery disease (CAD) is a type of cardiovascular disease manifested with myocardial infarction (MI), stable or unstable angina, and sudden death (Malakar et al., 2019). One of the major causes of death worldwide has been attributed to CAD (Lu et al., 2018). CAD results from the interaction of many risk factors, such as atherosclerosis, which is considered an important cause of CAD (Sarutipaiboon et al., 2020). Dyslipidemia, oxidative stress, and inflammation are involved in atherosclerosis (Sarutipaiboon et al., 2020).

Studies have shown more than 30% decrease in serum TC and LDL‐C changes in dietary intakes (Musaiger & Al‐Hazzaa, 2012). Several studies reported that minerals such as Cu, Zn, Mg, Se, and vitamins C and E have important functions in antioxidant and inflammation systems (Bonnefont‐Rousselot, 2004; Pavlovic & Sarac, 2011) and also have protective effects in preventing CAD (Wang et al., 2013). A review study indicates that diets rich in fruits and vegetables are associated with delayed atherosclerosis by high content of nonnutrient antioxidants such as polyphenolic compounds (Grassi et al., 2010). However, few studies examine the combined effects of dietary antioxidants such as vitamins, minerals, and phytochemicals based on indices such as DAI and DAQS as well as DPI that are considered as indices of dietary antioxidant status in turn single nutrients. Based on nutritional studies, DPI was calculated from the percentage of calories derived from foods rich in phytochemicals (McCarty, 2004), DAQS by summing the intake of certain antioxidant vitamins and minerals and assigning a quality value (Tur et al., 2005), and DAI by ranking individual intake compared with population mean (Wright et al., 2004).

A cross‐sectional study found that DAQS and DAI correlated inversely with IL‐1β and TNF‐α, but not with other inflammatory and oxidative stress factors (LuuHung, 2015). Another study on Iranian adults did not find any relation between high adherence to DAQS and dyslipidemia (Shahavandi et al., 2021).

Along with diet, genetic polymorphisms of some genes related to lipid levels and severity of artery stenosis affect the risk of CAD (Pollin & Quartuccio, 2013). (rs708272) Taq1B variant is placed in the first intron of the cholesterol ester transfer protein (CETP) gene on the long arm of chromosome 16q‐21 (Ordovas, 2000). A silent mutation of GUANINE base (G) to adenine base (A) created Taq1B (Drayna & Lawn, 1987). G base (frequent allele) with a cutting site for Taq1 endonuclease enzyme is labeled as B1 allele and A (less common allele) without Taq1 restriction site called as B2 allele (Hannuksela et al., 1994). Plasma CETP expression is influenced by Taq1B and regulates the transfer of cholesteryl esters among lipoproteins (Mirmiran et al., 2017). Many studies have indicated that Taq1B plays an important role in determining the size of serum lipoproteins and the severity of CAD, but the findings are inconsistent and contradictory (Lu et al., 2013; Rahimi et al., 2011; Rayat et al., 2022).

Based on current evidence, dietary components such as antioxidant vitamins, minerals, and phytochemicals may have some beneficial effects on blood vessels. However, to our best knowledge, there are limited studies that have examined the combined effects of dietary antioxidant components, such as vitamins and minerals with genes on vascular health. Current studies have investigated the association between genes and dietary components on health profiles. Therefore, this study aimed to evaluate the combined effect of adherence to dietary indices such as DAQS, DAI, and DPI with Taq1B on serum lipid levels and the severity of coronary artery stenosis in patients undergoing coronary angiography.

## 2 METHODS

### 2.1 Study design and participants

The number of participants in this study included 453 patients aged 35–75 years. Participants were referred for coronary angiography to Afshar Hospital in Yazd, Iran, from September 2020 to October 2021. Subjects were excluded if they had the following criteria: a history of cancer, myocardial infarction (MI), chronic heart failure (CHF), percutaneous coronary intervention (PCI), coronary artery bypass grafting (CABG), kidney failure, liver disease and use of its medications, certain perceptual or psychological disorders, immune system failure, acquired immunodeficiency syndrome (AIDS), extreme obesity (body mass index [BMI] >40 kg/m^2^), restriction in oral intake, pregnancy, and lactation. Finally, we included patients of both sexes, aged 35–75 years, who had the ability and willingness to participate in our study. Written informed consent was completed by participants. The Ethical Committee of Shahid Sadoughi University (SSU) of Medical Sciences Yazd, Iran, approved this study and gave an ethical code (IR.SSU.SPH.REC.1400.079).

### 2.2 DNA extraction and genotyping

**FIGURE 1:**
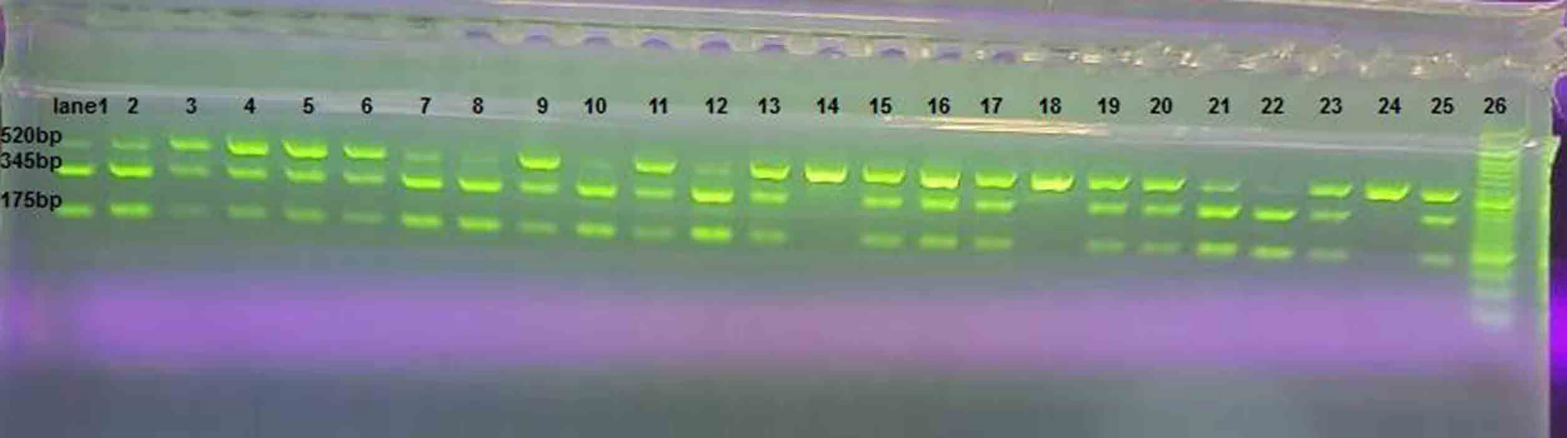
The digested fragments of the 708272‐CETP on 2% agarose gel electrophoresis. The ladder marker (lane 26) was 50 bp, the heterozygous B1B2 genotype had three bands of 175, 345, and 520 bp (lanes 1–13, 15–17, 19–21, 23, and 25), and the homozygous B2B2 genotype had one band of 520 bp (lanes 14, 18, and 24), as well as the homozygous B1B1 genotype had two bands of 175 and 345 bp (lane 22).

Genomic DNA was isolated from white cells of whole blood according to the protocol of a kit (Simbiolab, Iran). Extracted DNA was stored at −20°C until analysis. The TaqIB variant was genotyped by PCR‐RFLP method. Primers for amplifying TaqIB variant were 5′‐ACTAGCCCAGAGAGAGGAGTG‐3′ and 5′‐CAGCCGCACACTAACCCTA‐3′ (Sina colon, Iran), which were designed by priprimer software and previous studies. The PCR protocol was as below: one cycle of primary denaturation at 95°C for 5 min, followed by 40 cycles (95°C for 30 s, 66°C for 30 s, and 72°C for 30 s), and final extension at 72°C for 5 min. PCR products were electrophoresed on 2% agarose gel (SinaClon, Iran) and then were digested by endonuclease of the Taq1 (Cat No. ER0672) (Fermentase, Lithuania). The enzymatic digestion was performed in a final volume of 30 μL, containing 10 μL of PCR products, 17.5 μL of distilled water, 2 μL of buffer, and 0.5 μL of enzyme. The solution in the microtubes was mixed well by pipetting. Then, it was incubated at 37°C for 16 h in a water bath for enzymatic digestion. Digested fragments were electrophoresed on 2% agarose gel with a voltage of 100 V for 1 h. Taq1 recognizes a specific sequence of four to six base pairs and cleaves the sequence between the T and C nucleotides. Analysis of digested products on 2% agarose gel contained in three fragments: uncut homozygous B2B2 that had one band (520 base pair [bp]), cut heterozygous B1B2 with three bands (175, 345, and 520 bp), and cut homozygous B1B1 with two bands (175, 345 bp), is shown in Figure 1.

### 2.3 Assessment of lipid profile

Blood samples were obtained after an overnight fasting. The samples were stored in tubes containing EDTA. Serum was isolated by a centrifuge at 3634 *g* for 5 min, 4°C, and then stored at −80°C. Lipid parameters such as TG, HDL‐C, LDL‐C, and TC were measured using a commercial kit Pars Azmun (Tehran, Iran). The criteria for abnormal lipid levels were based on the National Cholesterol Education Program Adult Treatment Panel III (NCEP ATP III): TG ≥150 mg/dL and HDL‐C <40 mg dL for males, HDL‐C <50 mg/dL for females, LDL‐C ≥130 mg/dL, and TC ≥200 mg/dL (National Cholesterol Education Program (NCEP) Expert Panel on Detection, Evaluation, and Treatment of High Blood Cholesterol in Adults (Adult Treatment Panel III), 2002).

### 2.4 Assessment of coronary artery stenosis scores

The severity of coronary artery stenosis was examined through coronary angiographies. GS was assessed as described below: 1 point for ≤25% stenosis, 2 points for 26%–50%, 4 points for 51%–75%, 8 points for 76%–90%, 16 points for 91%–99%, and 32 points for full stenosis. In addition, the type and location of coronary artery stenosis were considered for calculating GS. A coefficient of 5 for the left main coronary artery, 2.5 for the left anterior descending and proximal, 1.5 for the mid‐segment of the left anterior descending coronary artery, 1 for the right proximal, and 0.5 for other segments. The final GS was obtained by summing stenosis scores and coefficients for each lumen (Avci et al., 2016; Gensini, 1983). GS ≥23 was considered as intermediate‐ to high‐risk severity of coronary artery stenosis and <23 low risk (İpek et al., 2017).

SS was calculated based on an online calculator version 2.0 (http://syntaxscore.org/calculator/syntaxscore/frameset.htm), using functional and anatomical parameters of the arteries with stenosis ≥50% and a diameter of ≥1.5 mm (İpek et al., 2017; Ryan, 1988). SS <22 was considered as low severity of coronary artery stenosis and SS ≥22 moderate–severe (İpek et al., 2017). The coronary angiographies were explained by experienced cardiologists who were blinded for some variables except for age and sex.

### 2.5 Anthropometric assessment

Trained nutritionists measured weight using a portable digital scale (Omron:BF511) with an accuracy of 100 g and height using a stature meter with an accuracy of 0.1 cm, as well as waist circumference (WC) by a nonstretchable tape with an accuracy of 0.5 cm. All measurements were assessed based on a standard protocol. We calculated body mass index (BMI) by dividing weight (kg) by the square of height (m^2^).

### 2.6 Assessment of dietary intake

The usual dietary intake of participants during the previous year was assessed using a 178‐item Food Frequency Questionnaire (FFQ), which was validated and found to be reliable by Zimorovat et al. (2020). Ten questions about the intake of Yazd‐specific food items such as Korno bread, dried bread, oil (Ardeh), and confectionery (Pashmak, Qotab, Nane Berenji, Haji badam, Loz, Baghlava, and cooked beets) were added to the previous questionnaire (Esfahani et al., 2010). Trained nutritionists collected the dietary data. Reported portion sizes from patients were converted to grams per day using household scales (Ghaffarpour et al., 1999). Nutrients were extracted using Nutritionist 4 software (First Databank Inc. Hearst Corp, San Bruno, CA).

### 2.7 Assessment of DAI, DAQS, and DPI

To calculate DAI based on the method by Wright et al. (2004), we standardized the intake of zinc, manganese, selenium, and vitamins C, E, and A derived from the FFQ by subtracting the global mean and dividing by the global standard deviation, and (SD) then, we summed the standardized intakes of the following as below:

$$\mathrm{DAI}=\sum_{i=1}^{n=6}\frac{\text{Indidual intake}-\text{Mean}}{\mathrm{SD}}$$

To create DAQS, we compared the daily intake of antioxidant nutrients including selenium, zinc, vitamin A, vitamin C, and vitamin E to the recommended dietary intake (RDI). Based on the method by Tur et al. (2005), we assigned a value of 0 to each nutrient when its intake was lower than two‐thirds of the RDI and a value of 1 when its intake was higher than two‐thirds of the RDI. We used the lower intake of two‐thirds of the RDI for each of these nutrients as the criterion to estimate the risk of inadequate intake (Aranceta et al., 2001).

We calculated the DPI according to the method by McCarty (2004) (DPI = [daily energy derived from phytochemical‐rich foods (kcal)/total daily energy intake (kcal)] × 100). We considered plant‐based dietary components, such as fruits, vegetables, legumes, whole grains, nuts, soy products, seeds, extra virgin olive oil, natural fruit and vegetable juices, and tomato sauces, as phytochemical‐rich foods. However, we did not include coffee, tea, and potatoes in this category (Farhangi et al., 2017; McCarty, 2004; Vincent et al., 2010).

### 2.8 Assessment of other variables

We evaluated daily physical activity using the International Physical Activity Questionnaire (IPAQ) (Moghaddam et al., 2012). Experienced nutritionists collected other data, including age, gender, smoking status, and medication use.

### 2.9 Statistical analysis

An Independent sample *t*‐test was used to compare continuous variables across DAQS, DAI, and DPI indices while a Chi‐square test was performed for categorical variables. The differences in abnormal HDL, LDL, TG, and TC, and high GS and SS among Taq1B genotypes and adherence of DAQS, DAI, and DPI indices were tested by one‐way ANOVA and ANCOVA analyses in crude and adjusted models, respectively. Two‐way ANOVA was used for interactions in both model crude and adjusted. Gender, BMI, smoking status, physical activity, and intake of energy were considered as confounders for adjusting. Pearson's *χ* ^2^ statistic was used for assessing the Hardy–Weinberg equilibrium (HWE). Statistical analyses were conducted using statistical package for the social sciences (SPSS) version 26.0 (IBM Corporation, USA). *p*‐Value <.05 was used as the significance level for all analyses, except for HWE, in which we used *p*‐value >.05.

## 3 RESULTS

The general and dietary intake characteristics of participants according to adherence to dietary indices of DAQS, DAI, and DPI are presented in Table 1. Smoking status and gender are distributed differently between patients with high‐ and low‐adherence DAQS, DAI, and DPI. BMI and physical activity differed significantly between those with high‐ and low‐adherence of DAI and DPI indices, respectively (*p* = .006, *p* = .004).

**TABLE 1:** Baseline characteristics and dietary intake based on adherence to DAQS, DAI, and DPI.

Variables	DAQS			DAI			DPI		
	Low ≤ 4	High > 4	*p*‐Value^a^	Low ≤ −1.08	High > −1.08	*p*‐Value^a^	Low ≤ 714.24	High > 714.24	*p*‐Value^a^
Gender, male, *n* (%)^b^	96 (52.2)	188 (69.9)	>.001	104 (46.0)	180 (79.3)	>.001	116 (51.3)	168 (74.0)	>.001
Age, year	57.67 ± 9.26	56.29 ± 9.42	.12	57.37 ± 9.37	56.34 ± 9.36	.24	56.74 ± 9.52	56.96 ± 9.24	.80
BMI, kg/m^2^	27.72 ± 4.57	27.21 ± 3.85	.21	27.96 ± 4.50	26.88 ± 3.71	.006	27.78 ± 4.37	27.06 ± 3.91	.06
Physical activity, *n* (%)^b^									
Sedentary	71 (38.6)	81 (30.7)	.21	88 (39.3)	64 (28.6)	.05	92 (41.3)	60 (26.7)	.004
Moderate	57 (31)	90 (34.1)		68 (30.4)	79 (35.3)		62 (27.8)	85 (37.8)	
Active	56 (30.4)	93 (35.2)		68 (30.4)	81 (36.2)		69 (30.9)	80 (35.6)	
Smoking status, *n* (%)^b^									
Nonsmoker	138 (75.0)	156 (58.0)	>.001	179 (79.2)	115 (50.7)	>.001	160 (70.8)	134 (59.0)	.01
Former smoker	8 (4.3)	9 (3.3)		8 (3.5)	9 (4.0)		10 (4.4)	7 (3.1)	
Current smoker	38 (20.7)	104 (38.7)		39 (17.3)	103 (45.4)		56 (24.8)	86 (37.9)	
Medicine consumption, yes *n* (%)^b^									
Statins	67 (36.4)	96 (35.7)	.87	91 (40.3)	72 (31.7)	.05	85 (37.6)	78 (34.4)	.47
Antidiabetic^b^	53 (28.8)	94 (34.9)	.17	78 (34.5)	69 (30.4)	.34	74 (32.7)	73 (32.2)	.89
Nutrient intake^a^									
Total energy (kcal/day)	1853.21 ± 647.12	3275.65 ± 1168.82	>.001	1826.94 ± 462.50	3564.98 ± 1100.30	>.001	2022.75 ± 797.17	3370.04 ± 1181.13	>.001
Carbohydrates, g/day	285.22 ± 105.89	497.79 ± 190.51	>.001	279.40 ± 78.23	542.92 ± 181.86	>.001	297.73 ± 118.08	524.67 ± 185.14	>.001
Proteins, g/day	68.59 ± 28.08	124.37 ± 53.77	>.001	66.47 ± 20.52	136.81 ± 51.70	>.001	76.16 ± 35.41	127.16 ± 54.95	>.001
Fats, g/day	51.77 ± 25.80	93.60 ± 43.61	>.001	51.86 ± 22.68	101.24 ± 43.67	>.001	59.69 ± 30.95	93.45 ± 46.00	>.001
MUFA, g/day	15.84 ± 7.77	28.12 ± 12.88	>.001	15.78 ± 6.45	30.45 ± 13.02	>.001	18.67 ± 10.33	27.57 ± 13.14	>.001
PUFA, g/day	12.37 ± 9.16	22.79 ± 13.22	>.001	11.77 ± 5.99	25.32 ± 14.14	>.001	13.90 ± 8.29	23.19 ± 14.69	>.001
SFA, g/day	15.68 ± 8.27	27.99 ± 15.04	>.001	16.23 ± 9.13	29.72 ± 14.93	>.001	18.43 ± 11.17	27.53 ± 15.20	>.001
Vitamin C (mg)^a^	138.11 ± 70.77	330.37 ± 192.65	>.001	148.27 ± 74.11	355.82 ± 197.18	>.001	148.43 ± 74.13	355.66 ± 197.35	>.001
Vitamin E (mg)^a^	11.25 ± 7.60	22.19 ± 12.21	>.001	10.83 ± 4.49	24.63 ± 12.88	>.001	12.92 ± 8.88	22.55 ± 12.50	>.001
Vitamin A (mg)^a^	462.50 ± 186.45	1077.95 ± 657.19	>.001	515.35 ± 204.37	1139.21 ± 697.73	>.001	562.99 ± 278.16	1091.78 ± 711.54	>.001
Zinc (mg)^a^	10.07 ± 4.29	18.19 ± 7.87	>.001	9.57 ± 2.82	20.19 ± 7.47	>.001	10.73 ± 4.45	19.04 ± 8.12	>.001
Selenium (μg)^a^	67.40 ± 28.70	121.10 ± 66.38	>.001	64.23 ± 18.56	134.19 ± 67.01	>.001	78.35 ± 48.28	120.13 ± 63.96	>.001
Manganese (mg)^a^	5.67 ± 2.54	8.54 ± 3.74	>.001	5.38 ± 1.80	9.36 ± 3.83	>.001	5.84 ± 2.91	8.90 ± 3.57	>.001

*Note*: The degrees of adherence to DAQS and DAI are categorized into two categories, “high” adherence and “low” adherence, based on the median intake.

Abbreviations: BMI, body mass index; DAI, dietary antioxidant index; DAQS, dietary antioxidant quality score; MUFA, monounsaturated fatty acid; PUFA, polyunsaturated fatty acid; SFA, saturated fatty acid.

^a^ Data presented as Mean ± SD.

^b^ Obtained from Chi‐squared test and independent *t*‐test for categorical and continuous variables, respectively.

Participants in the highest adherence to DAQS, DAI, and DPI compared with those in the lowest adherence had significantly higher intakes of energy, protein, carbohydrate, fat, polyunsaturated fatty acids (PUFA), monounsaturated fatty acids (MUFA), saturated fatty acids (SFA), vitamins C, E, and A, zinc, selenium, and manganese (*p* < .001).

The results of the frequency analysis of Taq1B genotypes showed that 10.4% were B1B1, 72.4% were B1B2, and 17.2% were B2B2. However, genotypes were not within HWE (*p* < .05 ).

### 3.1 Comparison of lipid profile and GS and SS between Taq1B genotypes in different adherences to DAQS, DAI, and DPI

GS, SS, and lipid profiles were compared among Taq1B genotypes in different adherences to DAQS, DAI, and DPI as shown in Tables 2, 3, 4, respectively. Patients with high adherence to DAQS, DAI, and DPI did not differ from those with low adherence in the distribution of HDL, LDL, TG, TC levels, and SS among Taq1B genotypes. In lower‐adherence DAQS, subjects with the B2B2 genotype had significantly lower GS compared with B1 allele carriers (*p* = .03), but this significance vanished after adjusting for confounders such as gender, smoking status, and energy.

**TABLE 2:** Interaction between the CETP TaqIB polymorphism and DAQS on the Gensini score, syntax score, and lipid profile.

Variables	Model crude			*p* ^a^	*p* ^c^	Model adjusted			*p* ^b^	*p* ^c^
	CETP Taq1B genotype					CETP Taq1B genotype				
	B1B1 (*n* = 47)	B1B2 (*n* = 328)	B2B2 (*n* = 78)			B1B1 (*n* = 47)	B1B2 (*n* = 328)	B2B2 (*n* = 78)		
	Mean ± SE	Mean ± SE	Mean ± SE			Mean ± SE	Mean ± SE	Mean ± SE		
HDL‐c (mg/day)										
Low	49.25 ± 3.20	49.65 ± 1.03	51.28 ± 2.12	.76	.93	49.46 ± 3.12	49.78 ± 1.03	50.68 ± 2.08	.91	.92
High	45.78 ± 1.78	47.59 ± 0.81	49.14 ± 1.78	.43		45.87 ± 1.98	47.52 ± 0.80	49.41 ± 1.69	.38	
LDL‐c (mg/dL)										
Low	96.62 ± 9.05	100.63 ± 3.37	100.19 ± 9.67	.94	.88	97.71 ± 10.85	100.58 ± 3.60	99.92 ± 7.24	.96	.87
High	86.55 ± 6.10	97.02 ± 2.76	96.88 ± 6.28	.35		86.68 ± 6.86	96.92 ± 2.79	97.22 ± 5.87	.37	
TC (mg/dL)										
Low	196.10 ± 37.22	204.98 ± 10.22	191.00 ± 14.33	.80	.74	198.59 ± 29.53	204.50 ± 9.81	191.84 ± 19.69	.84	.80
High	203.09 ± 23.01	196.53 ± 6.51	201.55 ± 18.07	.91		203.60 ± 17.96	196.48 ± 7.32	201.39 ± 15.38	.91	
TG (mg/dL)										
Low	148.10 ± 15.79	144.34 ± 6.10	161.24 ± 19.37	.54	.71	147.71 ± 20.56	144.92 ± 6.83	159.12 ± 13.71	.65	.71
High	179.47 ± 20.01	156.86 ± 5.29	166.54 ± 15.91	.34		178.77 ± 15.09	157.02 ± 6.15	166.32 ± 12.92	.37	
Gensini score										
Low	35.00 ± 10.23	37.20 ± 3.75	17.33 ± 3.86	.03	.01	36.18 ± 9.85	36.27 ± 3.27	20.50 ± 6.57	.10	.06
High	28.29 ± 7.71	34.37 ± 2.66	43.70 ± 7.81	.23		27.15 ± 7.00	35.02 ± 2.85	41.68 ± 5.99	.28	
Syntax score										
Low	10.66 ± 3.02	11.12 ± 1.06	6.75 ± 1.57	.15	.07	10.84 ± 2.91	10.85 ± 0.96	7.73 ± 1.94	.35	.18
High	8.49 ± 2.60	10.57 ± 0.86	13.15 ± 2.40	.28		8.26 ± 2.26	10.72 ± 0.92	12.68 ± 1.94	.33	

*Note*: The degrees of adherence to DAQS are classified into two categories, “high” adherence and “low” adherence, based on the median intake. *p*‐Values <.05 were considered significant.

Abbreviations: DAQS, dietary antioxidant quality score; HDL‐c, high‐density lipoprotein cholesterol; LDL‐c, low‐density lipoprotein cholesterol; TC, total cholesterol; TG, triglyceride.

^a^ *p* based on one‐way ANOVA.

^b^ *p* adjusted for gender, smoking status, and energy.

^c^ Obtained from two‐way ANOVA for both model crude and adjusted.

**TABLE 3:** Interaction between the CETP TaqIB polymorphism and DAI on the Gensini score, syntax score, and lipid profile.

Variables	Model crude			*p* ^a^	*p* ^c^	Model adjusted			*p* ^b^	*p* ^c^
	CETP Taq1B genotype					CETP Taq1B genotype				
	B1B1 (*n* = 47)	B1B2 (*n* = 328)	B2B2 (*n* = 78)			B1B1 (*n* = 47)	B1B2 (*n* = 328)	B2B2 (*n* = 78)		
	Mean ± SE	Mean ± SE	Mean ± SE			Mean ± SE	Mean ± SE	Mean ± SE		
HDL‐c (mg/day)										
Low	48.99 ± 2.72	49.37 ± 0.88	51.90 ± 2.04	.45	.74	48.94 ± 2.56	49.44 ± 0.91	51.65 ± 1.86	.53	.81
High	45.20 ± 1.82	47.50 ± 0.92	48.15 ± 1.76	.56		45.41 ± 2.26	47.44 ± 0.90	48.22 ± 1.87	.61	
LDL‐c (mg/dL)										
Low	91.75 ± 7.24	101.36 ± 2.95	101.51 ± 8.85	.59	.98	92.79 ± 8.94	101.46 ± 3.18	100.55 ± 6.49	.65	.98
High	88.15 ± 7.12	95.62 ± 3.09	94.97 ± 6.36	.66		88.60 ± 7.77	95.44 ± 3.10	95.41 ± 6.45	.71	
TC (mg/dL)										
Low	198.69 ± 27.87	203.14 ± 8.62	188.30 ± 12.47	.73	.66	202.68 ± 23.21	203.27 ± 8.27	185.68 ± 16.85	.64	.66
High	202.61 ± 27.46	196.83 ± 7.41	206.06 ± 20.69	.87		202.46 ± 21.09	197.10 ± 8.42	205.02 ± 17.52	.90	
TG (mg/dL)										
Low	160.92 ± 16.59	157.31 ± 5.89	164.02 ± 17.14	.89	.54	162.46 ± 17.77	157.97 ± 6.33	160.48 ± 12.90	.96	.48
High	176.35 ± 22.92	146.03 ± 5.41	164.45 ± 17.73	.14		176.97 ± 16.52	145.60 ± 6.59	165.86 ± 13.72	.12	
Gensini score										
Low	24.09 ± 7.78	34.18 ± 3.14	20.35 ± 4.24	.08	.10	26.07 ± 7.94	33.96 ± 2.83	20.21 ± 5.76	.08	.18
High	35.55 ± 9.16	36.91 ± 3.09	44.68 ± 8.67	.57		32.66 ± 8.08	37.58 ± 3.22	43.81 ± 6.71	.54	
Syntax score										
Low	6.71 ± 2.17	10.84 ± 0.99	7.36 ± 1.49	.11	.09	7.30 ± 2.53	10.78 ± 0.90	7.30 ± 1.84	.13	.15
High	11.18 ± 3.15	10.75 ± 0.90	13.51 ± 2.70	.50		10.60 ± 2.49	10.85 ± 0.99	13.49 ± 2.07	.49	

*Note*: The degrees of adherence to DAI are classified into two categories, “high” adherence and “low” adherence, based on the median intake. *p*‐Values <.05 were considered significant.

Abbreviations: DAI, dietary antioxidant index; HDL‐c, high‐density lipoprotein cholesterol; LDL‐c, low‐density lipoprotein cholesterol; TC, total cholesterol; TG, triglyceride.

^a^ *p* based on one‐way ANOVA.

^b^ *p* adjusted for gender, BMI, smoking status, and energy.

^c^ Obtained from two‐way ANOVA both model crude and adjusted.

**TABLE 4:** Interaction between the CETP TaqIB polymorphism and DPI on the Gensini score, syntax score, and lipid profile.

Variables	Model crude			*p* ^a^	*p* ^c^	Model adjusted			*p* ^b^	*p* ^c^
	CETP Taq1B genotype					CETP Taq1B genotype				
	B1B1 (*n* = 47)	B1B2 (*n* = 328)	B2B2 (*n* = 78)			B1B1 (*n* = 47)	B1B2 (*n* = 328)	B2B2 (*n* = 78)		
	Mean ± SE	Mean ± SE	Mean ± SE			Mean ± SE	Mean ± SE	Mean ± SE		
HDL‐c (mg/day)										
Low	48.14 ± 2.27	49.25 ± 0.89	51.11 ± 1.82	.56	.94	48.05 ± 2.32	49.27 ± 0.89	50.69 ± 1.87	.65	.99
High	45.33 ± 2.17	47.64 ± 0.91	49.14 ± 2.01	.48		45.72 ± 2.54	47.24 ± 0.90	49.24 ± 1.82	.47	
LDL‐c (mg/dL)										
Low	93.61 ± 6.41	103.86 ± 3.03	106.37 ± 9.48	.44	.87	95.56 ± 8.05	102.35 ± 3.24	106.84 ± 6.75	.56	.83
High	85.00 ± 8.10	93.22 ± 2.96	91.06 ± 5.81	.63		85.41 ± 8.28	92.90 ± 2.96	92.23 ± 5.93	.69	
TC (mg/dL)										
Low	196.15 ± 22.63	209.82 ± 9.01	203.90 ± 20.59	.84	.66	201.49 ± 22.82	208.35 ± 9.18	207.03 ± 19.14	.96	.81
High	206.68 ± 33.89	190.31 ± 6.90	190.68 ± 13.12	.75		206.42 ± 20.96	189.68 ± 7.50	192.76 ± 15.01	.75	
TG (mg/dL)										
Low	166.31 ± 14.81	153.37 ± 5.41	152.49 ± 12.63	.67	.46	166.00 ± 13.76	152.00 ± 5.54	151.48 ± 11.54	.62	.50
High	173.35 ± 27.46	150.06 ± 5.92	174.83 ± 20.34	.21		172.69 ± 20.71	150.19 ± 7.41	175.48 ± 14.83	.23	
Gensini score										
Low	31.19 ± 8.36	37.31 ± 3.35	25.54 ± 6.47	.28	.28	33.10 ± 7.91	36.27 ± 3.18	27.94 ± 6.63	.52	.44
High	29.49 ± 9.26	33.78 ± 2.87	38.22 ± 7.27	.68		25.99 ± 8.48	34.60 ± 3.03	37.49 ± 6.07	.53	
Syntax score										
Low	8.25 ± 2.22	11.18 ± 1.01	7.92 ± 1.60	.23	.21	8.71 ± 2.32	10.89 ± 0.93	8.56 ± 1.95	.44	.45
High	10.34 ± 3.60	10.42 ± 0.87	12.56 ± 2.55	.62		9.28 ± 2.76	10.58 ± 0.99	12.44 ± 1.98	.59	

*Note*: The degrees of adherence to DPI are classified into two categories, “high” adherence and “low” adherence, based on the median intake. *p*‐Values <.05 were considered significant.

Abbreviations: DPI, dietary phytochemical index; HDL‐c, high‐density lipoprotein cholesterol; LDL‐c, low‐density lipoprotein cholesterol; TC, total cholesterol; TG, triglyceride.

^a^ *p* based on one‐way ANOVA.

^b^ *p* adjusted for gender, physical activity, smoking status, and energy.

^c^ Obtained from two‐way ANOVA both model crude and adjusted.

### 3.2 Interaction between DAQS, DAI, and DPI and Taq1B variants on lipid profile and GS and SS

The interaction between Taq1B variant and DAQS, DAI, and DPI on serum lipid levels and coronary artery stenosis scores are shown in Tables 2, 3, 4, respectively. There was no significant interaction between Taq1B variants and DAQS, DAI, and DPI on TC, HDL, LDL, and TG levels and SS. There was a significant interaction between Taq1B variant and DAQS on GS (*p* = .01) in the unadjusted model, but this interaction became insignificant after controlling for confounders like gender, smoking status, and energy intake.

## 4 DISCUSSION

To our knowledge, this study is the first to examine how Taq1B polymorphism can be associated with lipid profile and coronary stenosis scores in relation to dietary antioxidant status indices in patients undergoing coronary angiography. In patients with high adherence to DAQS, DAI, or DPI, lipid profile, GS, and SS did not differ significantly by Taq1B genotypes. Also, Taq1B variant did not interact with high‐adherence DAQS, DAI, or DPI on lipid profile and GS or SS.

Few studies have investigated the association of DAQS and DAI with metabolic traits. Shahvandi et al. did not find any association between lipid profile and MetS in Iranian healthy adults with higher adherence to DAQS, although they reported improved systolic blood pressure (Shahavandi et al., 2021). A large case–control study in Iran showed that subjects with a DAI below the median had twice the odds of multiple sclerosis (MS) (Abdollahpour et al., 2022). Recently, a cross‐sectional study revealed an inverse association between DAI and depression and anxiety only in adolescent girls, in both unadjusted and adjusted models (Dehghan et al., 2022). More studies have been conducted on DPI than DAQS and DAI. Consistent with our findings, Firouzabadi et al. (2021) found no association between DPI and the odds of lipid profile abnormalities in both genders among Iranian adults. Similarly, a study did not show any relation between DPI and the change in lipid levels after 3 years of follow‐up (Zamani et al., 2020). In contrast, a cross‐sectional study found that a diet with higher DPI was linked to a lower risk of high blood pressure and metabolic syndrome (MetS), but not to other MetS‐related parameters (Vasmehjani et al., 2021). A recent review reported a negative association between DPI and hypertriglyceridemia, but not with other lipid parameters (Mehranfar et al., 2022). We did not find any relation between DAQS, DAI, and DPI and lipid profile, GS, or SS. Some factors that may explain the nonsignificant results in our study compared to others are sample sizes, type of study design, health status of cases, method of dietary assessment, absorption efficiency of nutrients and phytochemical compounds from the small intestine, and insufficient variation in antioxidants and phytochemicals in intakes above and below the median. Many studies have reported that Taq1B variant is associated with lipid profile and CAD, but there is still inconsistency (Kashani Farid et al., 2010; Lu et al., 2013; Rahimi et al., 2011; Raina et al., 2020). Other studies were in line with our findings as we did not find any relation in this aspect (Ghorban et al., 2015; Kalantar et al., 2022). We found that the relation between Taq1B and lipid levels and GS or SS did not change due to the interaction with the three dietary indices mentioned. We noticed that the B2 allele was more prevalent than the B1 allele (53% vs. 47%) in our study, which was similar to some studies in Asia, (Raina et al., 2020; Ramezani‐Jolfaie et al., 2020) but different from others (Kashani Farid et al., 2010; Lu et al., 2013; Rahimi et al., 2011; Rayat et al., 2022). Evidence shows that the B2 allele of Taq1B variant reduces CAD risk and increases HDL‐C levels by decreasing plasma CETP levels and activity (Boekholdt et al., 2005; Guo et al., 2016). In this line, we observed that subjects with B2B2 genotype and low‐adherence DAQS had lower GS than B1 allele carriers and there was an interaction between them, but this did not persist after adjustments. However, previous conflicting findings were explained by variations in the prevalence of the B2 allele across different locations and populations, which may account for the diverse results.

The only study that examined the interaction between Taq1B polymorphism and DPI had opposite results to ours, as Kalantar et al. (2022) detected an interaction between Taq1B polymorphism and DPI in the LDL/HDL ratio in type 2 diabetes patients. Our study's nonsignificant finding for interacting may be explained by the finding of Serafini et al. who showed that the antioxidant effect of other foods may be changed by consuming two different foods. No significant interaction between Taq1B polymorphism with adherence to Mediterranean diet (Med diet) on serum HDL‐C levels was found by Corella et al. (2010) in a sample of 4210 subjects with high CAD risk. It is possible to reduce the sample size in interaction studies caused random interactions. The variation in the results may depend on several factors, such as the number and health status of the participants (e.g., diabetes or CAD). Corella et al. (2000) also found that the Taq1B variant had a small effect on HDL‐C levels, explaining only 5.7% of the variation, this suggests that other genetic factors may influence HDL‐C levels as well. Moreover, dietary total antioxidant capacity (TAC), which reflects the overall antioxidant content of the diet, was associated with the severity of stenosis in patients with CAD and related risk factors, while the indices studied in the present study are only a part of dietary TAC.

This research has several strengths: It is the first study to investigate the interaction between Taq1B polymorphism and DAI or DAQS on some CAD‐related traits. We measured many confounders and adjusted for them in the statistical analysis. We used a validated and reliable FFQ to collect dietary data. We acknowledge some limitations of our study including despite measuring numerous variables, there may be some unmeasured variables that affect the antioxidant–outcome relationship. The cross‐sectional nature of the design could not establish causality for the insignificant association or interaction that we observed in our study. The neglect of the role of other SNPs and plasma CETP prevented us from interpreting the observed findings. Therefore, we suggest that similar studies should be conducted with minimal limitations as mentioned above.

## 5 CONCLUSION

The present study did not find any association or interaction between the three dietary indices and the Taq1B SNP on lipid profile levels and coronary artery stenosis scores. Insignificant results of this study provided a hypothesis for performing future research about the interaction between Taq1B SNP and dietary indices.

## AUTHOR CONTRIBUTIONS

**Azam Ahmadi Vasmehjani:** Data curation (supporting); formal analysis (equal); project administration (equal); writing – original draft (lead); writing – review and editing (equal). **Seyed Mostafa Seyed Hosseini:** Conceptualization (supporting); investigation (supporting); project administration (supporting); supervision (supporting). **Sayyed Saeid Khayyatzadeh:** Investigation (supporting); project administration (supporting). **Farzan Madadizadeh:** Formal analysis (lead). **Mahta Mazaheri‐Naeini:** Investigation (supporting); methodology (supporting); project administration (supporting). **Mahdie Yavari:** Investigation (supporting); methodology (supporting); project administration (supporting). **Zahra Darabi:** Methodology (supporting); project administration (supporting). **Sara Beigrezaei:** Methodology (supporting); project administration (supporting). **Marzieh Taftian:** Methodology (supporting); project administration (supporting). **Vahid Arabi:** Methodology (supporting); project administration (supporting). **Maryam Motallaei:** Methodology (supporting); project administration (supporting). **Amin Salehi‐Abargouei:** Conceptualization (lead); data curation (lead); investigation (lead); methodology (lead); project administration (lead); supervision (lead). **Azadeh Nadjarzadeh:** Conceptualization (lead); data curation (lead); investigation (lead); methodology (lead); project administration (lead); supervision (lead).

## FUNDING INFORMATION

Shahid Sadoughi University of Medical Sciences (SSUMS) supported this study.

## CONFLICT OF INTEREST STATEMENT

The authors have declared no competing interests.

## ETHICS STATEMENT

The Ethical Committee of Shahid Sadoughi University of Medical Sciences in Yazd approved the written informed consent (code number: 1400.079). All experiments were performed in accordance with the relevant guidelines and regulations. The present study obtained ethical approval from the Ethics Committee of Shahid Sadoughi University (SSU) of Medical Sciences Yazd, Iran (IR.SSU.SPH.REC.1400.079).

## CONSENT TO PARTICIPATE

The written informed consent was signed by all participants before the beginning of the study.

## Data Availability

The data and materials of the current study are available from the corresponding author on reasonable request.
